# Histone variant Htz1 promotes histone H3 acetylation to enhance nucleotide excision repair in Htz1 nucleosomes

**DOI:** 10.1093/nar/gkt688

**Published:** 2013-08-07

**Authors:** Yachuan Yu, Yanbo Deng, Simon H. Reed, Catherine B. Millar, Raymond Waters

**Affiliations:** ^1^Institute of Cancer & Genetics, School of Medicine, Cardiff University, Cardiff CF14 4XN, UK and ^2^Faculty of Life Sciences, Michael Smith Building, University of Manchester, Manchester M13 9PT, UK

## Abstract

Nucleotide excision repair (NER) is critical for maintaining genome integrity. How chromatin dynamics are regulated to facilitate this process in chromatin is still under exploration. We show here that a histone H2A variant, Htz1 (H2A.Z), in nucleosomes has a positive function in promoting efficient NER in yeast. Htz1 inherently enhances the occupancy of the histone acetyltransferase Gcn5 on chromatin to promote histone H3 acetylation after UV irradiation. Consequently, this results in an increased binding of a NER protein, Rad14, to damaged DNA. Cells without Htz1 show increased UV sensitivity and defective removal of UV-induced DNA damage in the Htz1-bearing nucleosomes at the repressed *MFA2* promoter, but not in the *HMRa* locus where Htz1 is normally absent. Thus, the effect of Htz1 on NER is specifically relevant to its presence in chromatin within a damaged region. The chromatin accessibility to micrococcal nuclease in the *MFA2* promoter is unaffected by *HTZ1* deletion. Acetylation on previously identified lysines of Htz1 plays little role in NER or cell survival after UV. In summary, we have identified a novel aspect of chromatin that regulates efficient NER, and we provide a model for how Htz1 influences NER in Htz1 nucleosomes.

## INTRODUCTION

The eukaryotic genome is packaged with histones and non-histone proteins to form chromatin. This characteristic genome organization in eukaryotes makes chromatin the primary platform for almost all DNA-based events, including replication, transcription and DNA repair (1–3). Hence, efforts have long been made to understand how chromatin impinges on genome organization and to decipher the epigenetic information encoded within chromatin. In addition to post-translational histone modifications (4) and ATP-dependent chromatin remodelling (5), incorporation of histone variants into nucleosomes (6,7) is another means by which cells regulate chromatin dynamics in response to internal and environmental cues.

The *Saccharomyces cerevisiae* Htz1 protein is a histone H2A variant that belongs to the highly conserved family of H2A.Z proteins (8). Sequence comparison indicates that H2A.Z proteins across species share more sequence identity with each other than with canonical histone H2A from the same species, suggesting that H2A.Z may have unique functions that are different from canonical histone H2A (9). Htz1 is incorporated into the nucleosomes in the form of a Htz1–H2B dimer by the ATP-dependent SWR complex to replace H2A–H2B, and the incorporation is independent of DNA replication (10–13).

The vast majority of *S. cerevisiae* promoters have a stereotypical chromatin architecture, characterized by two well-positioned nucleosomes flanking a nucleosome-depleted region (14–16). Genome-wide studies have shown that Htz1 is significantly enriched at the promoter-proximal nucleosomes of genes and in the euchromatin/heterochromatin boundaries (17–21). Acetylation at four lysines in the N-terminus is required for many of the functions of Htz1, including normal chromosome segregation (22) and maintenance of heterochromatin boundaries (23). At Htz1K14, the most abundantly acetylated site, acetylation is enriched at actively transcribed genes (24), and Htz1 acetylation is required for the efficient activation of *GAL1* (25) and oleate-induced genes (26). Reflecting these multiple roles, *htz1* alleles lacking the N-terminal acetylation sites have genetic interactions with genes involved in silencing, chromosome transmission, and transcription (27). Htz1 has also been linked to genome stability (28,29). The absence of Htz1 affects cell cycle progression and causes lethality or sickness in combination with checkpoint mutations (30). These results, together with the fact that *htz1Δ* cells are sensitive to some DNA-damaging reagents, suggest a role for Htz1 in maintaining genome integrity (12,22,23). Htz1 occupancy is dynamic near DNA strand breaks during double strand break repair (31–33), and Htz1 is necessary for strand resection and checkpoint activation at persistent DNA double-strand breaks (33). Other than this, the function of Htz1 in DNA repair remains largely unclear.

Nucleotide excision repair (NER) is a major and highly conserved repair pathway that cells use to remove a broad range of DNA damage, including cyclobutane pyrimidine dimers (CPDs) induced by UV irradiation (34). Compared with the vast efforts that have been made to comprehend the regulation of chromatin dynamics during transcription activation, studies on how NER operates in the chromatin environment are generally modest; therefore, our knowledge is limited. Early studies concentrated on the physical hindrance of nucleosomes on NER and revealed that lesions in the linker DNA and towards the ends of positioned nucleosomes are repaired faster than those in the centre of the nucleosomes (35–38). Meanwhile, a hypothetical model of ‘access-repair-restore’ was proposed, in an effort to rationalize the occurrence of chromatin remodelling during NER (39). More recently, direct evidence of roles for histone modifications and chromatin remodelling in NER has started to emerge. We previously showed that histone H3 acetylation by Gcn5 is activated in the repressed *MFA2* promoter after UV irradiation in *S. cerevisiae* (40,41). This UV-induced histone H3 hyperacetylation is necessary for the efficient repair of UV-induced CPDs in this locus. Rad16, a SWI/SNF-like NER protein responsible for the repair of DNA damage in non-transcribed DNA, governs the occupancy of Gcn5 on chromatin to regulate histone H3 acetylation after DNA damage (42,43). Others also showed that Dot1-controlled constitutive H3K79 methylation is required for the repair of CPDs in the *RPB2* and *HML* loci (44,45). In addition to histone modifications, chromatin remodelling by the SWI/SNF (46) and INO80 (47) ATP-dependent complexes was also found to be involved in the removal of CPDs from the transcriptionally silent *HML* locus by NER. Subunits of these chromatin remodelling complexes interact with Rad4-Rad23 in a UV dependent manner (46,47). The INO80 complex does not remodel the chromatin in the early stages after DNA damage. Instead, it is required for the restoration of chromatin after DNA damage is removed (47).

Here we examined how Htz1 contributes to the repair of UV-induced CPDs in the Htz1-containing nucleosomes in the *MFA2* promoter. Our data show that in wild-type cells, Htz1 is present in the two nucleosomes in the *MFA2* promoter, and Htz1 in these nucleosomes has a positive function for the efficient repair of UV-induced CPDs by NER. Deletion of *HTZ1* results in a significant reduction in the occupancy of Gcn5 on these two nucleosomes. This leads to a reduced response in histone H3 acetylation after UV. Consequently, the binding of Rad14 protein to damaged DNA is compromised, and the removal of CPDs is defective at *MFA2* in the absence of Htz1. However, deletion of *HTZ1* has no effect on histone H3 acetylation, the binding of Rad14 to damaged DNA and the repair of CPDs in the silent *HMRa1* locus where Htz1 is absent in wild-type cells bearing a functional *HTZ1* gene. This indicates a function for Htz1 in promoting NER in the Htz1-containing nucleosomes by regulating the occupancy of Gcn5 on chromatin, the UV-induced histone H3 acetylation and the binding of Rad14 to damaged DNA in the nucleosomes. Our results reveal a novel regulation of chromatin dynamics to ensure efficient repair of DNA damage.

## MATERIALS AND METHODS

### Yeast strains, growth, UV treatment, survival and repair

Yeast strains and plasmids are listed in Table 1. Yeast strains were grown in yeast complete medium, Yeast Extract-Peptone-Dextrose (YPD) at 30°C. To tag *GCN5* and *RAD14* with the Myc epitope, the plasmid p3747, a kind gift from Dr Richard A. Young, was used as a template to generate polymerase chain reaction (PCR) products containing the Myc epitope coding sequence and *URA3* selectable marker flanked by homologous regions designed to recombine at the 3′ end of the *GCN5* and *RAD14*, respectively. The PCR products were transformed into the BY4742, and its derivative *htz1Δ* mutant strain. Clones were selected for growth on uracil-minus plates. Insertion of the epitope coding sequence was confirmed by PCR and sequencing, and the expression of the epitope-tagged protein was confirmed by western blotting using an anti-Myc antibody. To generate pCM302, the *HTZ1* sequence was amplified, and cloned into the *Hind*III*–Sal*I sites of the yeast centromeric CEN/ARS, URA3 vector pRS416 (48). Point mutations were introduced into the *HTZ1* coding sequence using the QuikChange II Site-Directed Mutagenesis Kit (Stratagene) to generate pCM544 and pCM566. Mutations were verified by sequencing. For strains harbouring these plasmids, cells were grown in minimal medium without uracil. For UV survival experiments, cells at exponential phase (2–4 × 10^7^ cells/ml) from overnight culture were collected and resuspended in distilled water. Appropriate amount of cells were then spread onto YPD plates and subjected to various doses of UV treatment. Plates were kept in the dark at 30°C for 3–4 days before colonies were scored. For repair and post-UV chromatin immunoprecipitation (ChIP) experiments, cells at exponential phase (2–4 × 10^7^ cells/ml) were resuspended to a final concentration of 2 × 10^7^ cells/ml in pre-chilled phosphate buffered saline. The cell suspension was then either mock treated (without UV) or irradiated with 254 nm UV light at a dose of 100 J/m^2^. Mock-treated and irradiated cells were collected and resuspended in fresh YPD to allow for the indicated repair times in the dark at 30°C with shaking.

**Table 1.** Yeast strains

Strains	Genotype	Source
WT, BY4742	*MAT*α *his3Δ1* l*eu2Δ0 lys2Δ0 ura3Δ0*	Euroscarf
*htz1Δ*	*MAT*α *his3Δ1* l*eu2Δ0 lys2Δ0 ura3Δ0 htz1::* KanMX4	Euroscarf
*swr1Δ*	*MAT*α *his3Δ1* l*eu2Δ0 lys2Δ0 ura3Δ0 swr1::* KanMX4	Euroscarf
*yaf9Δ*	*MAT*α *his3Δ1* l*eu2Δ0 lys2Δ0 ura3Δ0 yaf9::* KanMX4	Euroscarf
*bdf1Δ*	*MAT*α *his3Δ1* l*eu2Δ0 lys2Δ0 ura3Δ0 bdf1::* KanMX4	Euroscarf
*gcn5Δ*	*MAT*α *his3Δ1* l*eu2Δ0 lys2Δ0 ura3Δ0 gcn5::* KanMX4	Euroscarf
WT, *HTZ1-*HA	*MAT*α *HHT1-HHF1Δ(HHT2-HHF2) leu2–3,112 ura3-52 lys2Δ201 HTZ1-*HA	Zhang *et al.*, 2005
WT, BY4742, *RAD14*-Myc	*MAT*α *his3Δ1* l*eu2Δ0 lys2Δ0 ura3Δ0 RAD14*::13*Myc-URA3*	This study
*htz1Δ, RAD14*-Myc	*MAT*α *his3Δ1* l*eu2Δ0 lys2Δ0 ura3Δ0 RAD14*::13*Myc-URA3 htz1:: NAT1*	This study
WT, BY4742, *GCN5*-Myc	*MATα his3Δ1 leu2Δ0 lys2Δ0 ura3Δ0 GCN5::18Myc-URA3*	This study
*htz1Δ, GCN5*-Myc	*MATα his3Δ1 leu2Δ0 lys2Δ0 ura3Δ0 htz1Δ*::kanMX4 *GCN5::18Myc-URA3*	This study
WT, W303	*MAT*α *ade2–1 leu2–3, 112 his3–1 ura3–52 trpl-100can1–100 HTZ1–3Flag-KanMX4*	Babiarz *et al.*, 2006
W303, *htz1K3,8,10,14R,*	*MAT*α *ade2–1 leu2–3, 112 his3–1 ura3–52 trpl-100can1–100 htz1K3, 8, 10, 14R-3Flag-KanMX4*	Babiarz *et al.*, 2006
pCM302	CEN6-ARS4 *URA3 HTZ1*	This study
pCM544	CEN6-ARS4 *URA3 htz1K3,8,10,14R*	This study
pCM566	CEN6-ARS4 *URA3 htz1K3,8,10,14Q*	This study

### Gel assay to analyse CPD repair in the overall genome

About 10 µg of DNA from each sample was treated with 5 µl of the CPD-specific endonuclease, obtained from *Micrococcus luteus*, for 1 h at 37°C, and then subjected to agarose gel electrophoresis (1.5%) under denaturing conditions (50 mM of NaOH, 1mM of EDTA in 1× Tris-acetate-EDTA [TAE] running buffer). The gel was washed, neutralized and stained with 1% ethidium bromide. The number of CPDs per kb DNA was calculated as described by Sutherland and Shih (49) and Bespalov *et al.* (50).

### Chromatin immunoprecipitation

ChIP was performed as described before (40). In brief, 50 ml of cells (2 × 10^7^ cells/ml) were cross-linked with 1.4 ml formaldehyde (37%) at room temperature for 20 min with shaking. Glycine (5.5 ml, 2.5 M) was added to stop cross-linking. Cells were collected, washed and then transferred to 2 ml Eppendorf tubes in 0.5 ml of ChIP buffer (50 mM HEPES-KOH, 140 mM NaCl, 1 mM EDTA, 1% Triton X-100, 0.1% sodium deoxycholate, 0.25% sodium dodecyl sulphate (SDS), 5 µl of protease inhibitor cocktail [Sigma UK, Cat No. P8215]). After addition of 0.5 ml of glass beads (425∼600 μm, Sigma), samples were vortexed at 4°C for 10 min. The cell lysate was collected for sonication. Sonication was carried out by using a Bioruptor (Diagenode) at 4°C, with power on ‘high’, 30 s on and 30 s off for 10 cycles to yield fragmented DNA of ∼300 bp in length. For experiments involving mononucleosomes, 300 μl of cell lysate in ChIP buffer after bead beating was supplemented with MgCl_2_ and CaCl_2_ to final concentrations of 20 and 5 mM, respectively, and then treated with 300 U of micrococcal nuclease (MNase; Nuclease S7, Roche) for 10 min at 37°C. The reaction was terminated by adding 1/50 volume of 0.5 M EDTA. The soluble MNase-fragmented chromatin was further homogenized by a brief sonication. Size analysis of purified DNA from samples after the treatment confirmed that only mononucleosomes are generated. For immunoprecipitation, 100 μl of sheared chromatin solution was incubated for 2 h at room temperature in a total volume of 1 ml in ChIP buffer containing specific antibodies. The amounts of antibodies used were 5 μg of anti-Htz1 (Abcam, ab4626), 2 μg of anti-HA (Upstate, 05–904), 5 μl of anti-histone H3 (Upstate, 17–10 046), 3 μg of anti-acetyl histone H3 (Upstate, 06–599; this antibody was raised against acetylated H3 at K9/K14) and 5 μg of anti-Myc (9B11, Cell Signalling technology, #2276). Fifty microlitres of protein G beads Sepharose (GE Healthcare Life Sciences) was added to the samples, and the mixture was incubated for another hour with shaking. The beads were washed, and DNA was eluted from the beads. After the cross-linking was reversed, DNA was finally purified using a Qiagen PCR purification kit. Quantitative PCR was performed in real time by using the iQ SYBR Green Supermix (Bio-Rad) in the Bio-Rad iCycler. Reactions were performed in triplicate for each sample, and melting curves were executed to ensure a single PCR product. For samples involving UV treatment, DNA damage was removed before PCR by using a PreCR DNA repair kit (New England Biolab). For mononucleosome ChIP, primers were designed to amplify the sequences within individual nucleosomes in the nucleosomes −2 and −1 in the *MFA2* promoter, and the nucleosomes 4 and 5 in the *HMRa1* locus. Otherwise, primers were designed to amplify sequences covering the above two nucleosomes in the *MFA2* and the *HMRa1* locus, respectively. Primer sequences are available on request.

### Analysis of CPDs at nucleotide resolution

This was carried out as previously described in detail (51). Repair analysis focused on both strands of the *Hae*III fragment (−517 to +83) in the *MFA2* promoter and the top strand of the *Rsa*I*-Bgl*II fragment (+61 to +476) in the *HMRa1* locus. The primers used were for detection of CPDs in the bottom strand of the *MFA2* promoter, 5′biotin-GATAGCTTTTTTCCCTCATCTATTTTCTCGGAAAACTTGGTG3′; for detection of CPDs in the top strand of the *MFA2* promoter, 5′biotin-GATAGCTTTTTTCCCTTGATTATATAGATTGTCTTTCTTTTCAGAGGAT3′; for detection of CPDs in the top strand of *HMRa1*, 5′biotin-GTAAGCTTTTTTTCATACGTTTATTTATGAACTACAAATTGT3′. Autoradiographs were obtained after scanning gels with a Typhoon Trio (GE Healthcare Life Sciences), and the signal for each band was quantified using ImageQuant 5.0 software. The repair rate was presented as the time needed to repair 50% of the initial damage (T_50%_). Bands close to each other in the gel and having the same repair rate were treated as one group. The data plotted in the graphs represent the average from at least three independent experiments. The statistical analysis to compare the repair rate between different strains was carried out using the student’s *t*-test.

### MNase sensitivity assay

Chromatin extraction and MNase treatment were performed as described previously (52). In brief, 400 ml of cells in 5 ml of lysis solution (1 M sorbitol, 5 mM β-mercaptoethanol) was treated with Zymolyase-20T (ICN Biochemicals) at a concentration of 25 mg per 1 g of wet cells for 30 min at 30°C. The spheroplasts were collected and lysed in 7 ml Ficoll solution (18% w/v Ficoll, 20 mM KH_2_PO_4_, pH 6.8, 1 mM MgCl_2_, 0.25 mM EGTA and 0.25 mM EDTA) by one stroke through a syringe. The nuclear pellets were collected, washed and resuspended in 1 ml of digestion buffer (15 mM Tris–HCl, pH 7.4, 75 mM NaCl, 3 mM MgCl_2_, 1.5 mM CaCl_2_, 1 mM β-mercaptoethanol). Two hundred fifty microlitres aliquots were treated with 0, 5, 15, 30 and 50 U of MNase (Nuclease S7, Roche) for 10 min at 37°C. The reaction was terminated by addition of 30 μl of stop solution to final concentrations of 1% SDS and 5 mM EDTA, respectively. DNA was purified, and digested with *Hae*III restriction enzyme. The detailed labelling procedures were as described previously (52). The primers used were the same as in the section of ‘analysis of CPDs at nucleotide resolution’ (see earlier section).

### Quantitative western blotting

Fifty millilitres of cells (2 × 10^7^ cells/ml) were lysed by beating with glass beads (425∼600 μm, Sigma) in 0.5 ml of dialysis buffer (20 mM HEPES-KOH, 10 mM MgSO_4_, 10 mM EGTA, 20% glycerol and 5 mM dithiothreitol, 5 μl of protease inhibitor cocktail [Sigma UK, Cat No. P8215]). The supernatant was collected for western blotting. Proteins were separated by 6% polyacrylamide–SDS gel electrophoresis. After transferring to the nitrocellulose membrane, the blots were probed with anti-Myc (9B11, Cell signalling, 2276) and anti-beta Actin (Abcam, ab8224) antibodies before with Cy5 conjugated anti-mouse IgG secondary antibodies (GE Healthcare Life Science, PA45009). Membranes were scanned via a Typhoon Trio (GE Healthcare) and images were quantified by ImageQuant software.

### Slot blot assay to analyse CPD levels

One hundred nanograms of DNA was denatured by adding NaOH to a final concentration of 0.4 M, and then transferred to a GeneScreen Plus nylon-based membrane via a slot-blot transfer apparatus (Bio-Dot Microfilitration Apparatus, Bio-Rad). The membrane was then probed with an antibody specific for CPDs (Anti-Thymine Dimer Clone KTM53, Kamiya Biomedical Company, Seattle). The detection was achieved via an ECF detection system (ECF western blotting reagent pack, GE Healthcare Life Sciences). The image was scanned with the Typhoon Trio (GE Healthcare Life Sciences), and quantified by ImageQuant 5.0 software.

## RESULTS

### Cells lacking the *HTZ1* and *SWR1* genes show increased UV sensitivity and slower removal of CPDs from the overall genome

**Figure 1.**
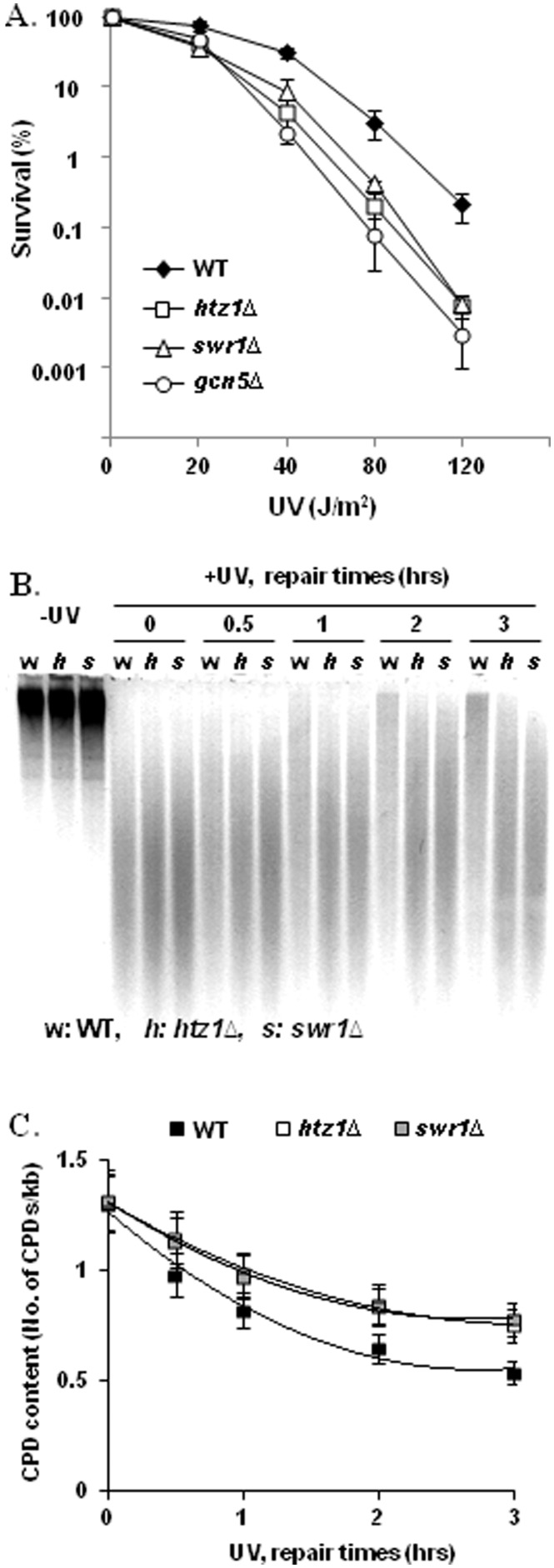
Htz1 is required for cell survival after UV and global CPD repair in the overall genome. (**A**) The UV sensitivity of wild-type cells and of cells lacking the *HTZ1*, *SWR1* or *GCN5* genes. Data are the average of at least three independent experiments ± SD. (**B**) Repair of CPDs from the genome. DNA from samples taken before and after UV (100 J/m^2^) was treated with a CPD-specific endonuclease. The digestion products were then separated by gel electrophoresis under denaturing conditions and visualized with EtBr. Here, 0, 0.5, 1, 2 and 3 indicate repair times (hours). (**C**) CPD content in genomic DNA. The gel in (B) was scanned using a Typhoon Trio (GE Healthcare Life Sciences), and the median migrating distances of the DNA smear were calculated using ImageQuant 5.0. CPD content (CPDs/kb) was calculated as described by Sutherland and Shih (49) and Bespalov *et al.* (50). The data were fitted to a second-order polynomial. Values represent the mean ± SD of three independent experiments.

Htz1 is incorporated into the nucleosomes by the SWR complex of which Swr1 is the catalytic subunit (10–12). Both of the *htz1Δ* and *swr1Δ* cells are sensitive to the DNA-damaging agent methyl methanesulfonate, which induces DNA strand breaks (12,22). We first tested whether deletion of *HTZ1* or *SWR1* will result in increased sensitivity to UV irradiation. We also included the *gcn5Δ* mutant for comparison. The *gcn5Δ* mutant is moderately sensitive to UV, and Gcn5 controls UV-induced histone H3 acetylation in the *MFA2* promoter, which is required for efficient repair of CPDs via NER (40,53). As shown in Figure 1A, both the *htz1Δ* and *swr1Δ* cells are indeed more sensitive to UV than the wild type. This prompted us to further investigate whether the increased UV sensitivity in the *htz1Δ* and *swr1Δ* mutant cells is related to less-efficient repair of UV-induced CPDs via NER. We used a gel-based assay to assess the repair of CPDs via NER in the total genomic DNA. Genomic DNA from samples taken before and at various times after UV irradiation (100 J/m^2^) was treated with a CPD-specific endonuclease and subjected to agarose gel electrophoresis under denaturing conditions. Recognition and then cleavage of damaged DNA at CPD sites by the enzyme generates fragmented DNA. The repair of CPDs in DNA by NER restores DNA to longer fragments that run more slowly in the gel. Figure 1B shows a representative gel examining the restoration of DNA towards longer fragments due to NER during a 3 h repair period; it indicates that the restoration of high molecular weight DNA occurs more slowly in the *htz1Δ* and *swr1Δ* mutants, compared with that in the wild type. The gel was also densitometrically scanned, and the CPD content in each sample was calculated based on a method described by Sutherland and Shih (49) and Bespalov *et al.* (50). The quantitative results (Figure 1C) show that the *htz1Δ* and *swr1Δ* mutants have more CPDs in their genome than the wild type does after 3 h repair time, indicating deletion of *HTZ1* or *SWR1* results in a defect in the repair of UV-induced CPDs from a considerable portion of the genome.

### Acetylation of Htz1 at K3, 8, 10, 14 does not contribute to UV survival and CPD repair

**Figure 2.**
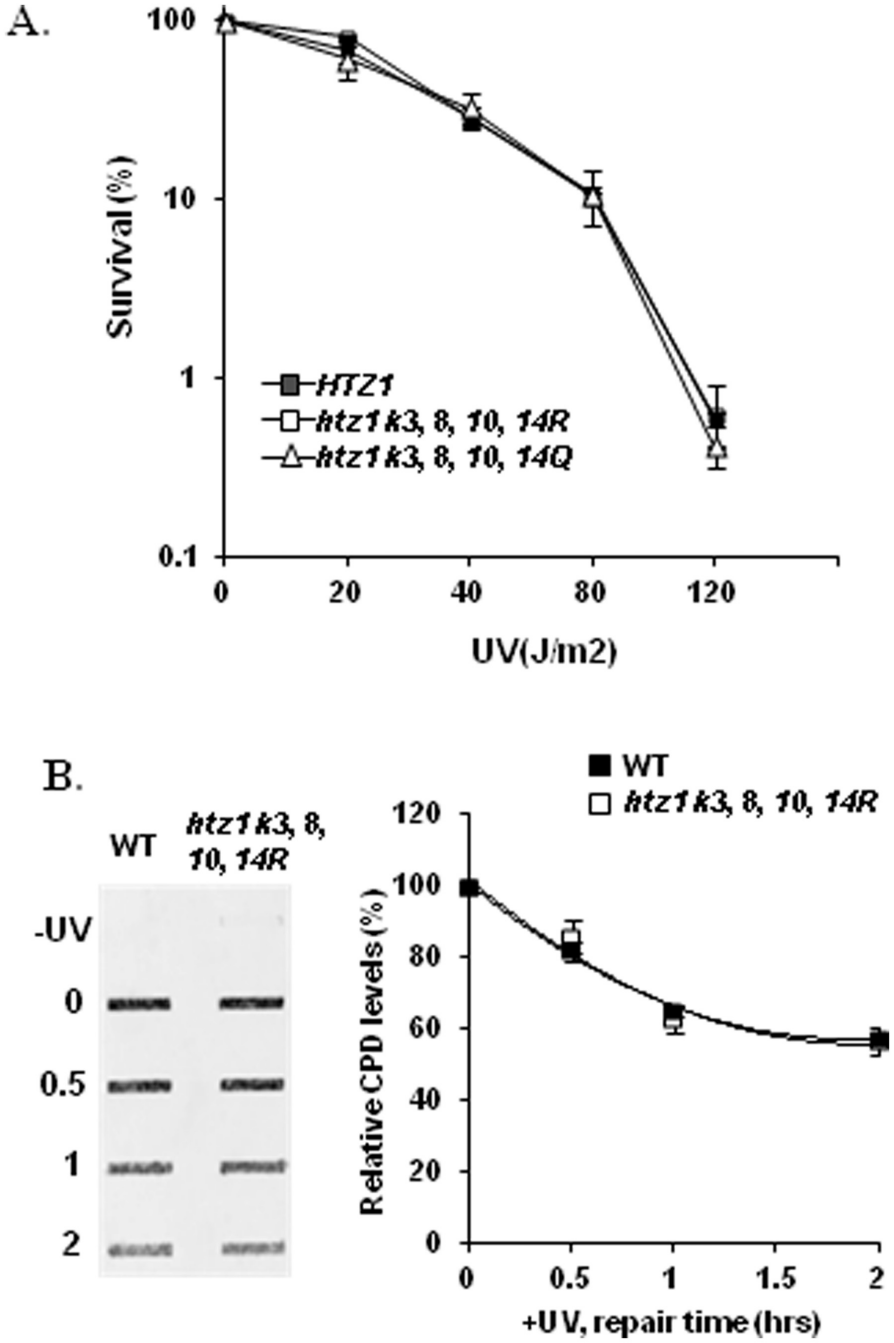
Htz1 acetylation at sites K3, 8, 10, 14 is not required for UV survival and CPD repair. (**A**) The sensitivity to UV irradiation of strains with wild-type *HTZ1* or *htz1* alleles in which the 4 N-terminal acetylation sites have been substituted by arginine or glutamine residues. Data are the average of at least three independent experiments ± SD. (**B**) CPD levels in the genome were detected by the slot-blot assay. DNA from samples taken after UV (100 J/m^2^) was probed with CPD-specific antibodies. Indicated on the left side of the image are the repair times in hours. CPD levels are presented relative to the amount in samples taken immediately after UV irradiation. Data were the average of at least three independent experiments ± SD, and fitted to a second-order polynomial.

Both Htz1 and Gcn5 are important for cell survival after UV irradiation. As removal of Gcn5 also affects the acetylation of Htz1 (23,24), one explanation could be that acetylation of Htz1 is important for cell survival and CPD repair after UV irradiation. To test this, we generated yeast centromeric plasmids carrying wild-type *HTZ1*; *htz1 K3, 8, 10, 14R*; or *htz1 K3, 8, 10, 14Q* alleles, and expressed them in the *htz1Δ* strain. In the *htz1 K3, 8, 10, 14R* plasmid, the four acetylatable lysines are replaced by arginines (R), which mimic the unacetylated state, whereas in the *htz1 K3, 8, 10, 14Q* plasmid, the four lysines were mutated to glutamines (Q), which mimic acetylated lysines. As indicated in Figure 2A, strains with these three plasmids showed the same sensitivity to UV. We also measured the content of CPDs in genomic DNA over a period of 2 h after UV treatment in cells harbouring wild-type *HTZ1* and *htz1 K3, 8, 10, 14R* plasmids by a slot-blot assay with antibodies against CPDs. The result indicates that there is no difference in CPD content between cells with wild-type *HTZ1* and the mutated *htz1 K3, 8, 10, 14R* (Figure 2B). To ensure that the phenomenon we observed with these strains is not due to plasmid expression, we tested cell survival and CPD repair from the overall genome after UV irradiation in a different wild type (W303) and its isogenic mutant in which all the four lysines of the endogenous Htz1 were mutated to arginines (R). These two strains again showed the same survival rate and repair of CPDs after UV irradiation (Supplementary Figure S1). Therefore, we conclude that acetylation at these sites does not contribute to CPD repair by NER and cell survival after UV irradiation.

### The absence of Htz1 reduces NER at the repressed *MFA2* promoter, but not at the *HMRa1* locus

**Figure 3.**
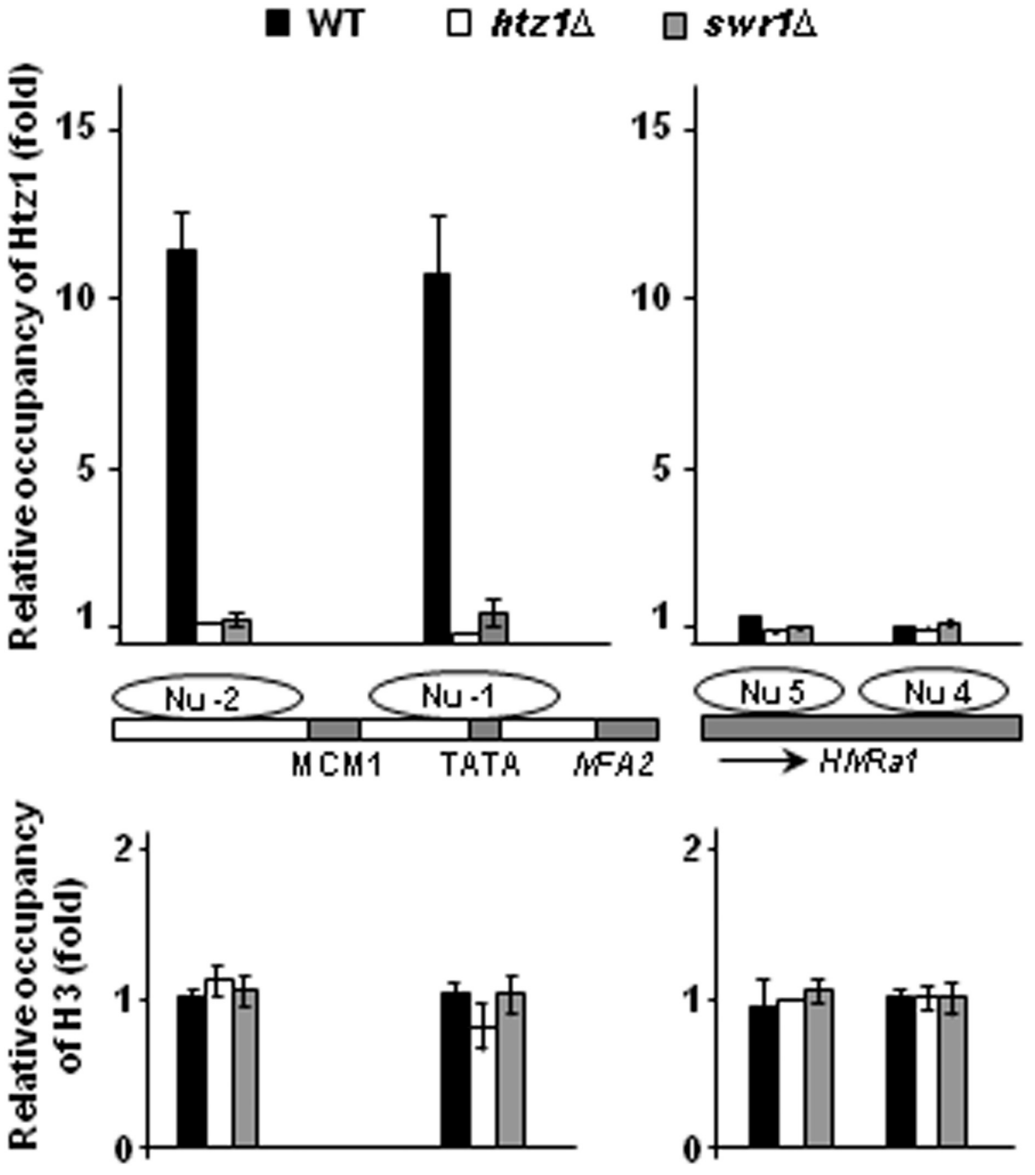
Htz1 occupies the *MFA2* promoter, but not the *HMRa1* inner sequence, in a Swr1-dependent manner. Mononucleosome ChIP analysis of Htz1 and H3 occupancy was performed using anti-Htz1 and anti-H3 antibodies. Gene structures are illustrated in the middle of the graph. Shaded areas are binding sites for regulatory factors and gene bodies. Htz1 occupancy is presented as the relative levels to that in the *htz1Δ* mutant, and H3 levels were normalized against that at Nu 5 in the *HMRa1* locus in the *htz1Δ* mutant. Data are the average of at least three independent experiments ± SD.

The data thus far show that Htz1, but not its acetylation, is required for NER. The results of the global repair assay in Figure 1B and C could either indicate a partial defect in the capability of the whole NER machinery or a defect in the repair of UV-induced CPDs after UV irradiation in certain regions, e.g. the Htz1 bearing nucleosomes, of the genome. Therefore, we further investigated the repair of CPDs at specific genetic loci that are either enriched or depleted for Htz1. To identify and select these, we examined available genome-wide data of Htz1 occupancy (20), and we chose two loci for further investigation: the *MFA2* promoter (nucleosome -2 and -1) (52) and the inner sequence (nucleosome 4 and 5) in the *HMRa1* locus (54). Both *MFA2* and *HMRa1* are transcriptionally inactive in our strains (α mating type), and have well-positioned nucleosomes. Mononucleosome ChIP using anti-Htz1 antibodies confirmed that Htz1 occupies the two nucleosomes in the repressed *MFA2* promoter, but it is absent from the *HMRa1* locus in wild-type cells bearing a functional Htz1 gene (Figure 3). As expected, deletion of *SWR1* reduces the presence of Htz1 in the repressed *MFA2* promoter to almost the level of that in the *htz1Δ* mutant cells. The occupancy of histone H3 remains the same in *MFA2* and *HMRa1*, and deletion of *HTZ1* and *SWR1* does not influence this.

**Figure 4.**
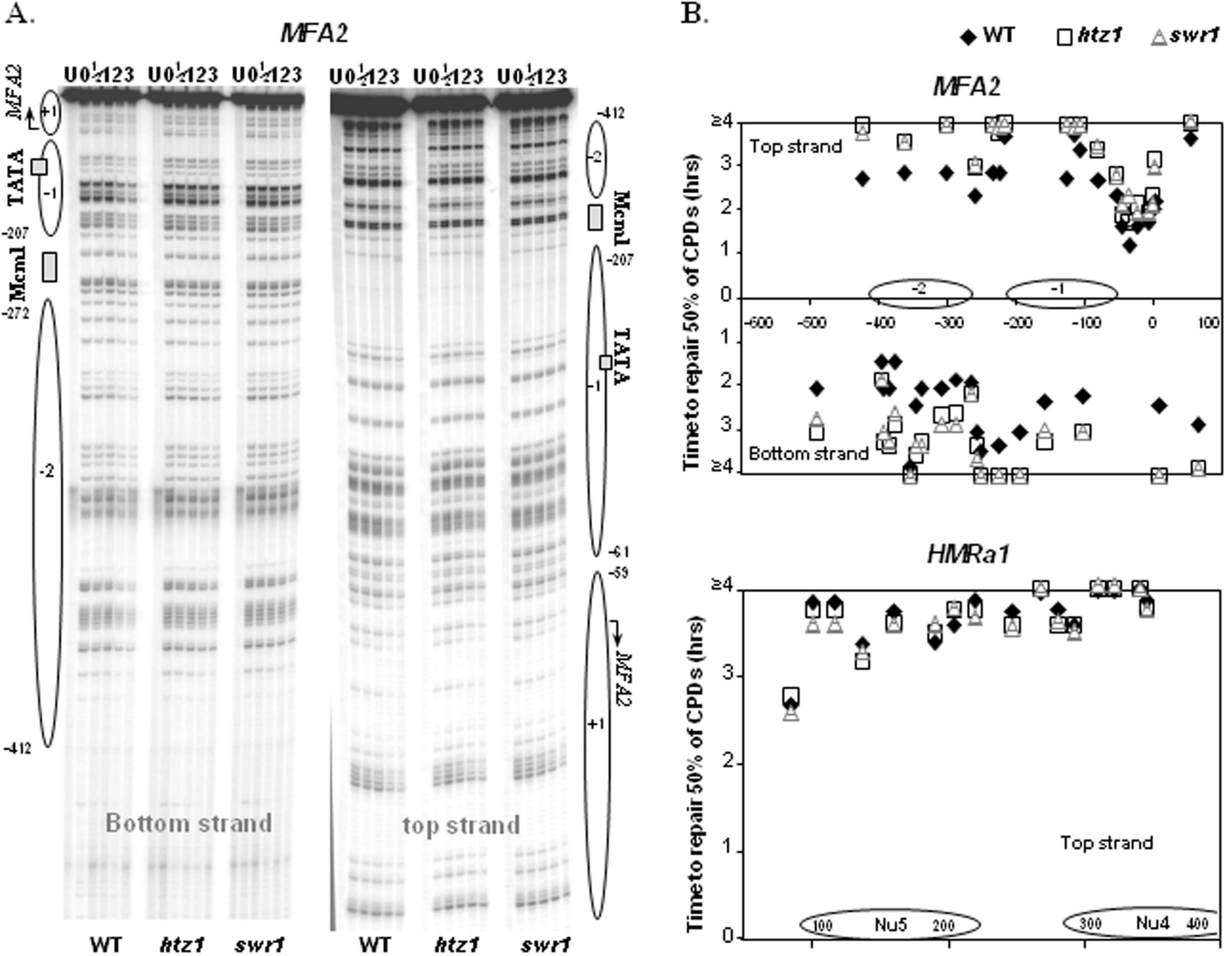
Deletion of *HTZ1* or *SWR1* reduces the repair of CPDs in the *MFA2* promoter. (**A**) Gels depicting CPDs in the top and bottom strands of a *Hae*III restriction fragment (−516 to +83) in the *MFA2* promoter after a UV dose of 100 J/m^2^. Lane U is DNA from mock-irradiated cells, while 0, 1/2, 1, 2 and 3 are DNA from irradiated cells after 0, 1/2, 1, 2 and 3 h repair, respectively. Alongside the gels are symbols representing nucleosome positions at *MFA2*. Nucleotide positions are allocated in relation to the *MFA2* start codon. (**B**) Time to remove 50% of the initial CPDs (T_50%_) at given sites. T_50%_ of a single CPD or a group of CPDs with a similar repair rate was calculated (T_50%_ < 3 h) or extrapolated (T_50%_ > 3 h). The T_50%_ of slowly repaired CPDs (≥4 h) was shown at the same level (≥4 h) on the graph. Data are the average of three to five independent experiments.

We next investigated how deletion of *HTZ1* and *SWR1* affects the repair of CPDs after UV irradiation in the *MFA2* and the *HMRa1* loci. Typical gels for the high resolution CPD repair analysis in the *MFA2* promoter are shown in Figure 4A. The gels for CPD repair in the *HMRa1* locus are shown in Supplementary Figure S2. Repair efficiency is quantitatively presented in Figure 4B as the time needed to remove 50% of initial CPDs (T_50%_) at specific sites. In the *MFA2* promoter, the repair of CPDs is significantly reduced at almost all sites across the two original Htz1-bearing nucleosome region, including the linker, in the *htz1Δ* and *swr1Δ* mutant cells compared with that in the wild-type cells (*t*-test, WT versus *htz1Δ*, *P* = 2.39 × 10^−5^, WT versus *swr1Δ*, *P* = 8.44 × 10^−5^). There is no evidence that the repair difference only occurs in certain regions within the two nucleosomes. Meanwhile, the repair of CPDs in these two nucleosomes in the *htz1Δ* and *swr1Δ* mutant cells are similar (*t*-test, *P* = 0.71). However, in the *HMRa1* locus where we analysed the repair in one strand, the repair of CPDs in the original non-Htz1 nucleosomes is equally efficient in all of these three strains (*t*-test, WT versus *htz1Δ*, *P* = 0.31; WT versus *swr1Δ*, *P* = 0.33; *htz1Δ* versus *swr1Δ*, *P* = 0.54). This clearly indicates that deletion of *HTZ1* or *SWR1* influences the repair of CPDs in the Htz1-containing nucleosomes in the *MFA2* promoter, but not in the non-Htz1 nucleosomes in the *HMRa1* locus. This cannot be attributed to a down-regulation of NER because repair of CPDs at *HMRa1* remains unchanged in *htz1Δ* cells. Therefore, Htz1 in the nucleosomes in wild-type cells has a direct effect on NER at the *MFA2* promoter.

### Depletion of *HTZ1* does not change the MNase sensitivity of DNA in chromatin in the *MFA2* promoter

**Figure 5.**
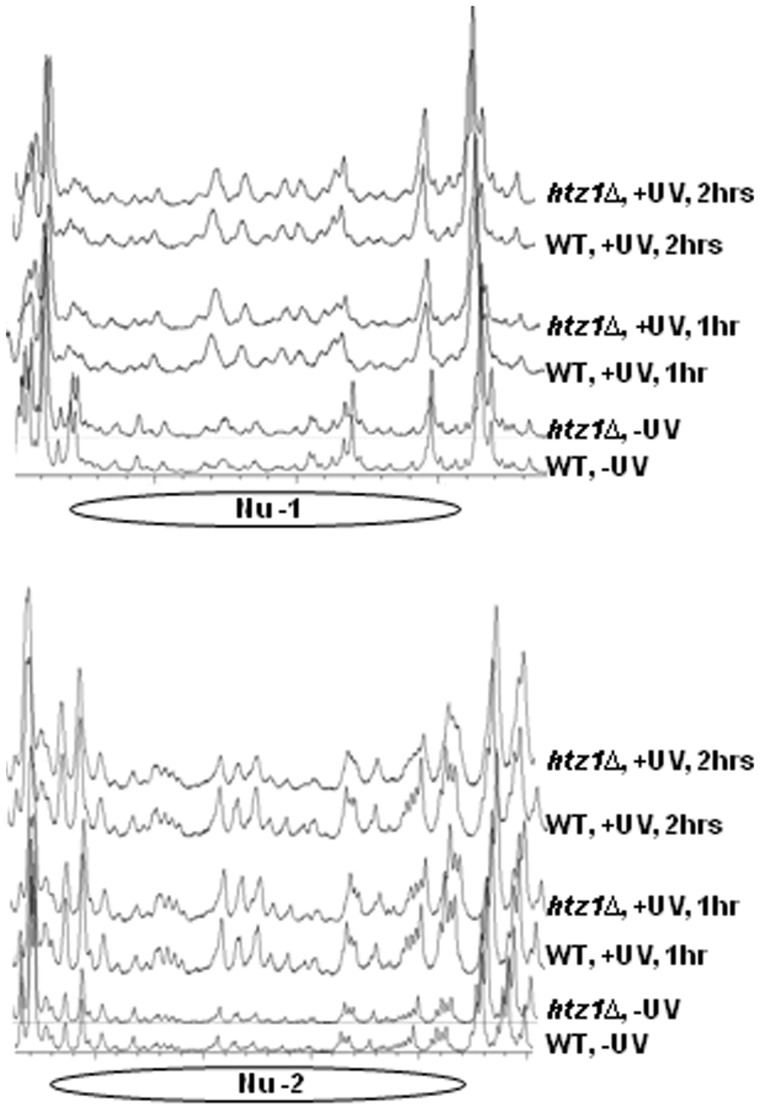
Deletion of *HTZ1* does not change the relative sensitivity of the nucleosomal DNA to MNase in the *MFA2* promoter. The graphs were obtained after scanning the lanes with 5 U of MNase in gels for the top and bottom strands of *MFA2* as shown in Supplementary Figure S3. The peaks indicate MNase-sensitive sites, which increase in size and frequency after UV treatment but are not different between wild-type and the *htz1Δ* cells. This is a representative of at least three independent experiments.

Incorporation of Htz1 into the nucleosomes could potentially change their stability, and also their susceptibility to chromatin remodelling when required. Both the default chromatin structure and chromatin remodelling after UV have a significant influence on CPD repair in the *MFA2* promoter (40,43). To address whether deletion of *HTZ1* results in any changes in chromatin accessibility in the *MFA2* promoter, and whether the deletion influences chromatin remodelling at this locus after UV treatment, we used a high-resolution nucleosome mapping approach (52) to examine the susceptibility of DNA in chromatin in the *MFA2* promoter to MNase digestion in wild-type and the *htz1Δ* cells. This method is capable of assessing the sensitivity to MNase digestion of every site in a DNA sequence of interest in chromatin, and it has single nucleotide resolution. All gels are presented in Supplementary Figure S3. We scanned these gels to produce graphs of MNase sensitivity at individual cutting sites within the chromatin, and presented them in Figure 5. These graphs reveal that MNase digestion in the *MFA2* promoter is almost identical between chromatin from wild-type cells and the *htz1Δ* cells; firstly, wild-type and the *htz1Δ* cells have the same nucleosome positioning patterns in the *MFA2* promoter; secondly, DNA within the two original Htz1 nucleosomes in the *MFA2* promoter is similarly sensitive to MNase in these two strains under normal growth condition; thirdly, DNA within the two nucleosomes in these two strains has similar degrees of increase in MNase sensitivity after UV irradiation (Figure 5). We observed the same extra peaks and peak heights within the nucleosomes in UV 1 h and UV 2 h samples irrespective of the presence of Htz1. Thus, we conclude that deletion of *HTZ1* does not measurably change the accessibility of the nucleosomal DNA in the *MFA2* promoter, nor does it influence the kinetics of chromatin becoming more sensitive to MNase due to chromatin remodelling in this region after UV treatment.

### Htz1 promotes the occupancy of Gcn5 and histone H3 hyperacetylation in Htz1 nucleosomes in the *MFA2* promoter after UV irradiation

**Figure 6.**
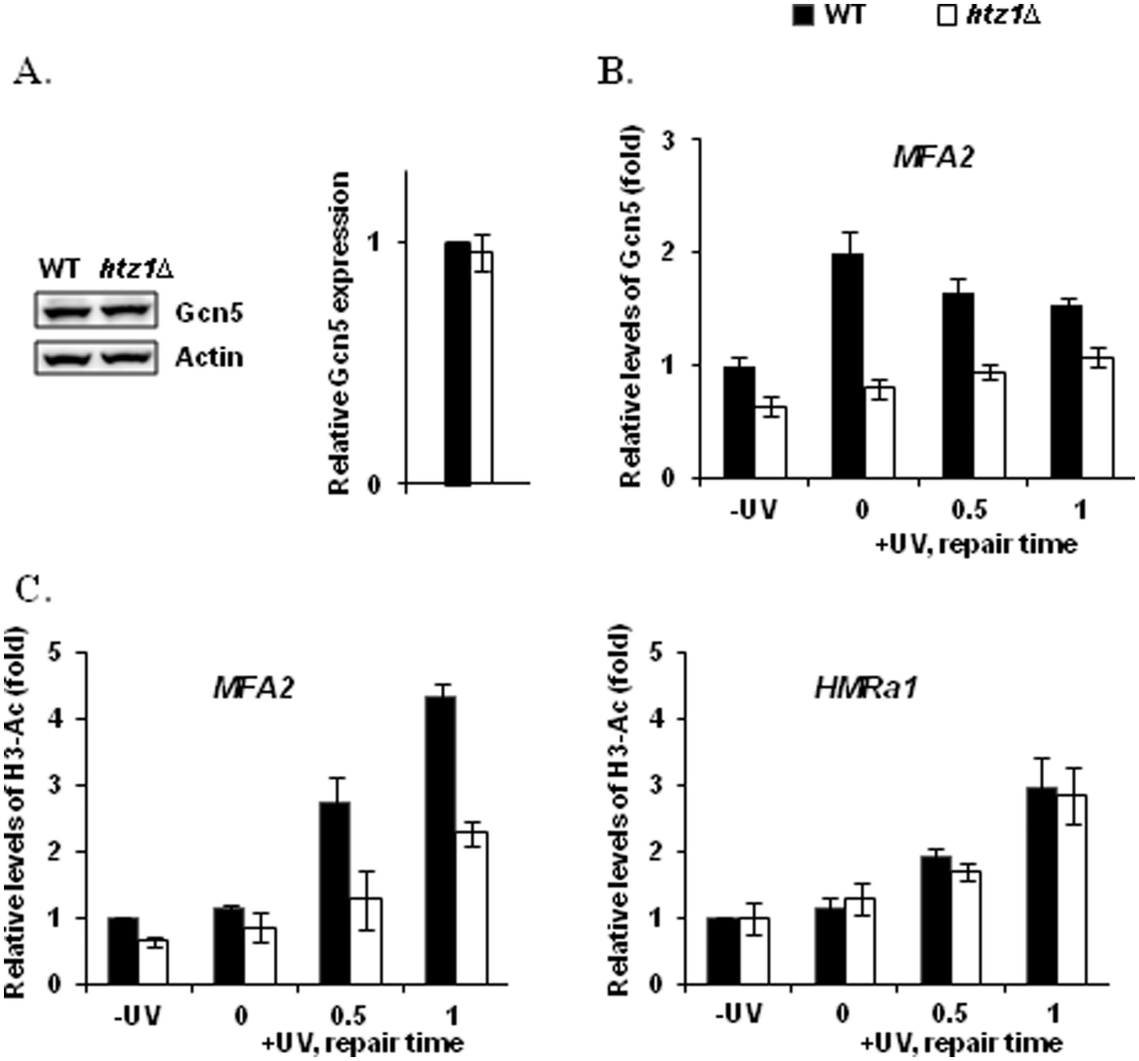
The occupancy of Gcn5 and induction of H3 acetylation at the *MFA2* promoter is partially dependent on Htz1. (**A**) Gcn5 expression in wild-type and the *htz1Δ* mutant. Gcn5 was detected with anti-Myc antibodies and Cy5 conjugated anti-mouse IgG secondary antibodies in *GCN5*-myc strains. The expression levels were normalized against those of Actin. (**B**) ChIP analysis of the occupancy of Gcn5 was performed with anti-Myc antibodies in *GCN5*-myc strains. The levels of Gcn5 binding at *MFA2* was normalized against those at the *HMRa1* locus, and then presented as the fold change relative to the mock irradiated sample in wild type. (**C**) ChIP analysis of Histone H3 acetylation (H3-Ac) was performed using Ac-H3 (K9, K14) antibodies. The H3 acetylation levels were first normalized against the H3 levels, and then presented as the fold change relative to the mock irradiated sample in wild type. −UV: mock irradiated samples; 0: cells received 100 J/m^2^ of UV without repair; 0.5 h repair or 1 h repair: cells were irradiated with UV and then were allowed to repair in YPD for the number of hours indicated. Data are the average of at least three independent experiments ± SD.

Our data on MNase sensitivity and repair indicate that deletion of *HTZ1* reduces the efficiency in repair of CPDs in the Htz1-containing nucleosomes in the *MFA2* promoter without causing major detectable changes in chromatin accessibility both before and after UV treatment. This is similar to what we observed with the *gcn5Δ* strain that also exhibits reduced NER at the repressed *MFA2* gene (40). In our previous reports (43) we showed that Rad7 and Rad16 proteins mediate the increased occupancy of the histone acetyltransferase Gcn5 on the nucleosomes at the *MFA2* promoter after UV irradiation, and this leads to histone hyperacetylation at H3K9/K14 which is required for efficient repair of UV-induced CPDs (40,43). Hence, we now focused our investigation on how Htz1 influences the occupancy of Gcn5 on chromatin and histone H3 acetylation by performing ChIP assays. We first measured the total expression level of Gcn5 protein in wild-type and the *htz1Δ* mutant by quantitative western blot. Our data indicated that the expression levels of Gcn5 remain the same in these two strains (Figure 6A). For ChIP assays, to ensure efficient amplification by PCR of DNA with UV-induced damage, we included a repair procedure in which DNA damage in all immunoprecipitated DNA was removed by a mixture of DNA repair enzymes (see ‘Materials and Methods’ section) before PCR. The levels of Gcn5 occupancy at the two nucleosomes in the *MFA2* promoter were normalized against those in the *HMRa1* locus. This reflects the binding of Gcn5 at *MFA2* in relation to that at *HMRa1*. The histone H3 acetylation levels at *MFA2* and *HMRa1* were normalized against the H3 levels at these loci, respectively. At *MFA2*, we observed an increase in the occupancy of Gcn5 in the wild-type cells after UV treatment as we did previously [Figure 6B (43)]. Deletion of *HTZ1* results in a reduction in the occupancy of Gcn5 at this locus both before and after UV treatment when compared with that at the *HMRa1* locus. Although the levels of Gcn5 occupancy are slightly increased in the *htz1Δ* cells after UV treatment, they are still significantly lower than that seen in wild type (Figure 6B). In relation to this, the *htz1Δ* mutant cells exhibit a lower constitutive level of histone H3K9/K14 acetylation (0.65 ± 0.07 in *htz1Δ* versus 1 in WT) in the promoter of *MFA2* before UV irradiation than in the wild-type cells (Figure 6C). More importantly, the increase in the levels of H3K9/K14 acetylation after UV irradiation in the *htz1Δ* mutant cells is significantly less than that in the wild-type cells (e.g. 2.28 ± 0.17 fold increase in *htz1Δ* versus 4.34 ± 0.22 fold increase in WT, 2 h after UV). In the *HMRa1* locus where Htz1 is normally absent, an increase in histone H3K9/K14 acetylation after UV treatment was also observed. Deletion of *HTZ1* has no influence on the extent of the increase (Figure 6C). To conclude, our data indicate that histone H3 acetylation at K9/K14 is activated at both the *MFA2* and *HMRa1* loci after UV, and specifically Htz1 in the nucleosomes of the *MFA2* promoter promotes the occupancy of the histone acetyltransferase Gcn5 on chromatin and histone H3K9/K14 acetylation on these nucleosomes.

### Htz1 promotes the binding of Rad14 to damaged DNA at the MFA2 promoter after UV

**Figure 7.**
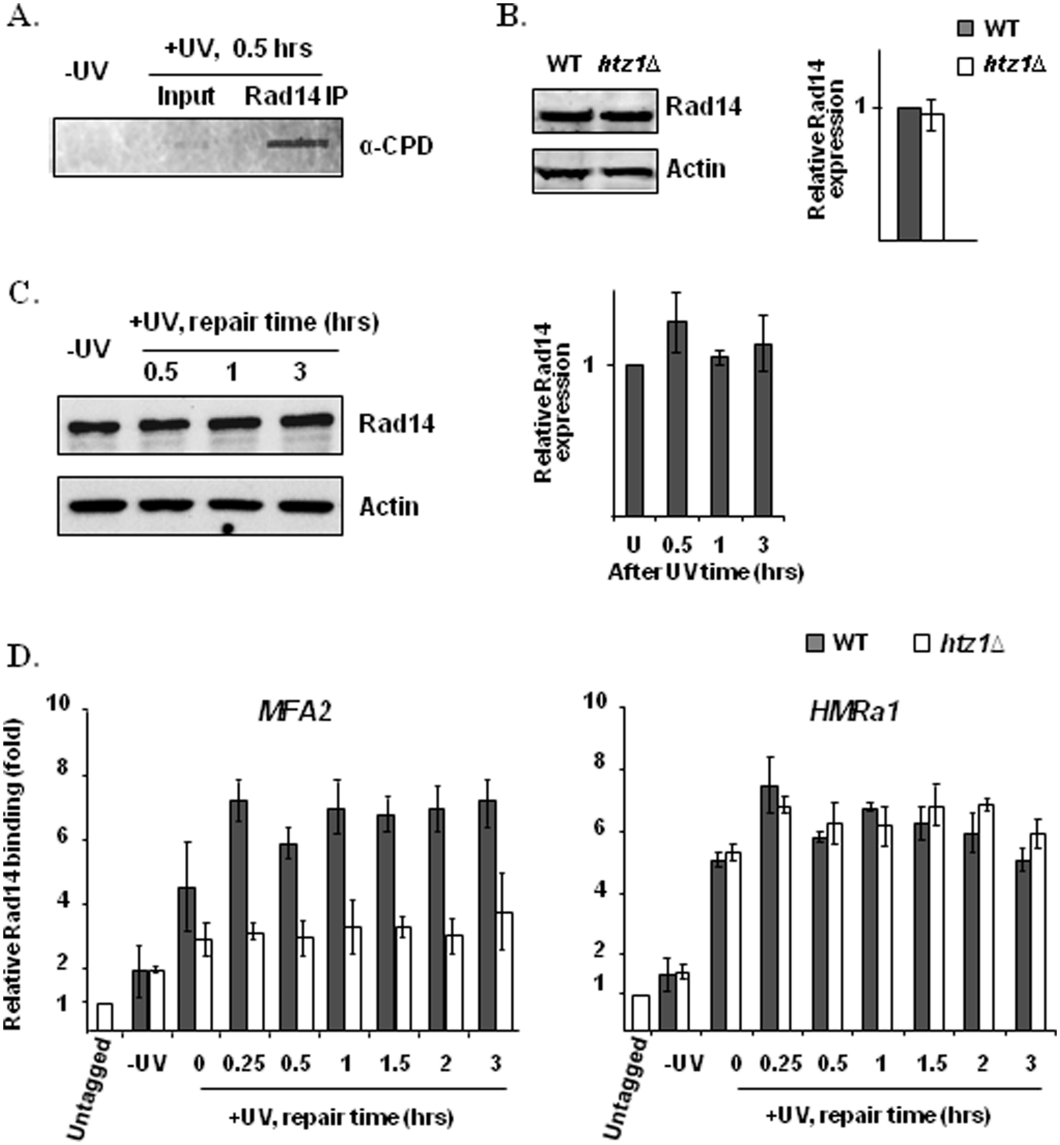
Rad14 binding to damaged DNA after UV treatment is enhanced by Htz1. Rad14 immunoprecipitation was performed with anti-Myc antibodies in *RAD14*-myc strains. Quantitative expression of Rad14 was determined with anti-Myc antibodies and Cy5 conjugated anti-mouse IgG secondary antibodies in *RAD14*-myc strains. (**A**) CPD content in DNA (10 ng) from UV mock treated, UV treated and Rad14 immunoprecipitated samples, as detected by the slot-blot assay. The amount of DNA was measured by a Nanodrop. (**B**) Rad14 expression in wild-type and the *htz1Δ* cells. (**C**) Rad14 expression in wild-type cells before and after UV irradiation. The quantitative levels of Rad14 expression in (B) and (C) were normalized against those of Actin. (**D**) The binding of Rad14 to the *MFA2* promoter and the *HMRa1* inner sequence. In (D), the level of Rad14 binding is present as the fold change relative to those from the untagged strain. Quantitative data are the average of at least three independent experiments ± SD.

The presence of Htz1 in the nucleosomes in the *MFA2* promoter helps to promote the increased occupancy of Gcn5 and histone H3K9/K14 acetylation in this region after UV treatment. These events are linked to a more efficient NER. We hypothesized that Htz1-associated histone H3 acetylation status is critical for the binding of core NER factors to damaged DNA in nucleosomes, and this binding decides the rate of lesion removal. To test this, we investigated how deletion of *HTZ1* influences the binding of a key NER factor, Rad14, to damaged DNA in the Htz1 nucleosomes at *MFA2* after UV. Rad14 is believed to act as a DNA damage recognition factor in the early steps of NER (55). After UV treatment, Rad14 bound DNA showed enriched CPDs (Figure 7A), confirming that Rad14 is preferentially associated with damaged DNA after UV irradiation. Quantitative western blotting showed that deletion of *HTZ1* does not change the expression level of Rad14 (Figure 7B), and the expression of Rad14 is unaffected by UV treatment (Figure 7C). However, in ChIP assays in which the immunoprecipitated DNA was again treated with the DNA repair enzyme kit before PCR, we observed a significant reduction in Rad14 binding to the original Htz1 nucleosomes at the *MFA2* promoter in the *htz1Δ* cells compared with that in the wild-type cells, while the binding of Rad14 at the *HMRa1* locus was not affected (Figure 7D). This result suggests a positive function for Htz1 in Htz1-containing nucleosomes to promote the binding of Rad14 to damaged DNA in chromatin after UV irradiation. We also noticed that Rad14 binds to both the *MFA2* promoter and the *HMRa1* inner sequence even before UV treatment (Figure 7D, comparing −UV samples with untagged samples), but this binding is intensified after UV irradiation. The increased binding of Rad14 to damaged DNA occurs so fast that we observed a significant increase in Rad14 binding to both loci immediately after UV irradiation (Figure 7D, 0 repair time). We did not observe any reduction of Rad14 binding to both loci over the repair time up to 3 h. At the UV dose used, there is still DNA damage that needs to be repaired after 3 h, and therefore the continued enhanced binding of Rad14 to DNA is expected.

## DISCUSSION

Emerging evidence suggests that efficient repair of DNA damage in the chromatin environment requires histone modifications and chromatin remodelling (56,57). Our previous studies, using the *S. cerevisiae MFA2* gene as an example, revealed that histone H3 acetylation and chromatin remodelling are activated after UV irradiation, and both events are required for NER to efficiently process UV-induced CPDs in chromatin (40). The UV-stimulated H3 acetylation is achieved via increased occupancy of Gcn5 at *MFA2*, which is regulated by the global genome NER (GG-NER) complex Rad7/Rad16/Abf1 (42,43). These data have shed considerable insight in how GG-NER operates on repressed chromatin, and they have led us to propose a model to explain how chromatin dynamics are regulated to permit NER (43). In the absence of UV histone H3 acetylation is maintained at a basal level, and chromatin remains in a closed structure at the promoter of *MFA2*. After UV irradiation, Rad7 and Rad16 promote the occupancy of Gcn5 at this promoter, resulting in elevated levels of histone H3 acetylation. This promotes an open chromatin structure at *MFA2*. The GG–NER complex has DNA translocase activity, but, unlike some SWI/SNF superfamily complexes, it is unable to slide nucleosomes (58). This is critical, as it prevents transcription initiation sites being exposed to transcription initiation factors during the process of repair. In this way, the GG–NER complex can promote specific changes in chromatin structure required for efficient GG-NER, while preventing unregulated gene transcription at the same time.

New features in chromatin structure and their implications in the relevant biological events have been rapidly identified. H2A.Z (Htz1 in *S. cerevisiae*) nucleosomes are found in many organisms, and these nucleosomes have particular functions that cannot be replaced by canonical H2A nucleosomes (59). Here, we found that deletion of *HTZ1* has a significant influence on the repair of UV-induced CPDs. By performing the same analysis in the Htz1-containing nucleosomes at the *MFA2* promoter and the non-Htz1-containing nucleosomes in the *HMRa1* locus, our experiments revealed that this defect is directly related to the absence of Htz1 in the nucleosomes. This implicates intrinsic properties of Htz1 nucleosomes in facilitating more efficient NER in these nucleosomes in wild-type cells. The positive functions of Htz1 nucleosomes influence NER in two ways. First, Htz1 promotes the occupancy of the histone acetyltransferase Gcn5 and therefore the UV-induced histone H3 acetylation in the Htz1-containing nucleosomes at *MFA2*. Second, Htz1 promotes the efficient binding of Rad14 to damaged DNA in the Htz1-bearing nucleosomes, thereby promoting NER after UV irradiation. These events are not mutually exclusive, and it is likely that the former enables the latter. This study has revealed an additional layer of information in chromatin at the *MFA2* promoter that cells use to regulate chromatin dynamics to ensure efficient DNA damage repair after UV irradiation.

Htz1 promotes NER in the Htz1-containing nucleosomes by either inherently influencing nucleosome stability or by its specific residues serving as modification sites for the binding of other molecules. Acetylation of Htz1 at one or more sites at lysines 3, 8, 10 and 14 is important for multiple functions of Htz1 (22–25,27). However, our data indicate that acetylation at these sites plays no roles in enabling more efficient NER and in influencing cell survival after UV. Others have also reported that point mutations at these sites do not recapitulate the sensitivity to the DNA-damaging agent methyl methanesulfonate that one sees with the *htz1Δ* mutant (22,23). This indicates that when cells encounter DNA-damaging agents, Htz1 has more functions in maintaining genome stability than those related to its N-terminal acetylation.

Incorporation of Htz1 into the nucleosomes to replace canonical histone H2A could possibly affect the nucleosome stability, higher order chromatin structure and their susceptibility to chromatin remodelling. However, available data failed to reach an exclusive conclusion regarding these issues. Various *in vitro* and *in vivo* studies gave contradictory results, with evidence being reported that the H2A.Z nucleosome is either more (18,60–62) or less stable (20,63,64) than the canonical H2A nucleosome. Our high-resolution MNase assay, which is capable of assessing the sensitivity of many sites in chromatin to MNase *in vivo* revealed that deletion of *HTZ1* has no effect on the accessibility of nucleosomal DNA to MNase in the original Htz1 nucleosomes at the repressed *MFA2* promoter. This is consistent with the available studies regarding the effect of Htz1 on chromatin structure (18,65). In addition, our assay also revealed that, with or without Htz1, these nucleosomes at *MFA2* all become more sensitive to MNase and to similar extents in response to UV irradiation. This suggests that using this assay Htz1 has no influence on chromatin remodelling in this region after UV irradiation. Hence the positive function of Htz1 in NER cannot be simply explained by the general stability of the nucleosomes.

On the other hand, our data indicate that these Htz1 nucleosomes display a unique feature in that Htz1 promotes the binding of Gcn5 to chromatin and hence histone H3 hyperacetylation in these nucleosomes after UV irradiation, and this acetylation is required for efficient NER (40). For NER to process DNA damage in repressive chromatin, for example, in the repressed *MFA2* promoter, NER proteins need to get access to DNA damage in chromatin. This may be facilitated by histone H3 acetylation and chromatin remodelling. Our Rad14 binding data showed that deletion of *HTZ1* reduces the binding of Rad14 to damaged DNA by 50% after UV in the original Htz1 nucleosomes in the *MFA2* promoter, but not in the *HMRa1* locus where Htz1 is absent in wild-type cells with a functional *HTZ1* gene. This is an indication of a unique function of Htz1 in the nucleosomes in promoting the binding of an important repair protein, Rad14, to damaged DNA in the nucleosomes after UV. It is directly reflected in the reduction in NER at *MFA2* in the absence of Htz1. Although Rad14 is a damaged DNA-binding protein (55) and there is no evidence so far indicating Rad14 interacts with histones, the presence of Htz1 may produce a favourable environment after UV, for example, through helping achieve an optimal level of histone H3 acetylation, to facilitate the enhanced binding of Rad14 to damaged DNA. Recently, two studies revealed a similar phenomenon in mammalian cells in processes other than NER (66,67). That is, H2A.Z promotes histone H4 acetylation and in combination with H4 acetylation, Htz1 promotes the recruitment of downstream effector proteins to chromatin. These events occur during transcription activation (66) and DNA double strand break repair (67). Taking all these data together, it suggests that this function of Htz1 (H2A.Z) may be conserved from yeast to humans, and that it has roles in different processes.

Our data revealed an intrinsic property of Htz1 in regulating chromatin modifications to facilitate NER, and this function is confined to Htz1 containing nucleosomes. Htz1 is only present in a portion of the nucleosomes in the yeast genome (20,21). CPDs in the canonical H2A nucleosomes are also repaired after UV. We provided data for one of those regions, namely, the *HMRa1* inner sequence, to indicate this. The question as to whether Htz1-containing nucleosomes mark regions for faster repair of CPDs by NER genome-wide can only be answered by entire genome studies using either ChIP-on-chip or ChIP seq approaches, and on which we are currently embarking.

**Figure 8.**
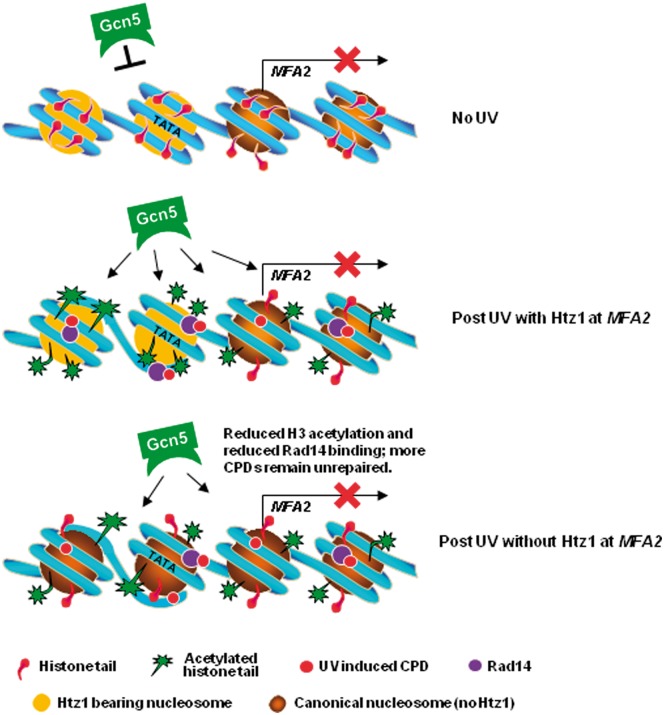
Model for chromatin remodelling at *MFA2* during NER. Top panel. In the absence of UV histone H3 tails remain unacetylated and chromatin remains repressive. Middle Panel. After UV irradiation, the increased occupancy of Gcn5 that is mediated via the Rad7/Rad16 GG-NER complex is promoted by the presence of Htz1. This helps achieve enhanced acetylation levels on histone H3, which are critical for the specific chromatin setting required for optimal NER, so ensuring maximal binding of repair proteins, e.g. Rad14, to damaged DNA. This leads to efficient lesion removal. Lower Panel. Without Htz1 in these nucleosomes, the occupancy of Gcn5 and the acetylation levels on histone H3 are reduced. Therefore, the maximal binding of repair proteins to damaged DNA cannot be achieved and more CPDs remain unrepaired.

This study, in addition to our previous ones (40,42,43), demonstrates that multiple elements at the nucleosome level in chromatin decide the dynamics of chromatin after UV irradiation at a single genomic location, namely *MFA2*. They serve to facilitate efficient removal of DNA damage by NER. Based on these data, we extend our model (43) on how chromatin is regulated for efficient repair of UV-induced DNA damage, and summarize it in Figure 8 to include the novel role for Htz1 described in this article. After UV irradiation, Rad7 and Rad16 regulate the occupancy of Gcn5 at the *MFA2* promoter to acetylate histone H3. Htz1 in the nucleosomes in this region promotes and maintains increased binding of Gcn5 to the nucleosomes, thus resulting in increased acetylation levels on histone H3. This is critical for the required setting of a specific chromatin structure for efficient NER. The increased levels of H3 acetylation enhance the recruitment of Rad14, and possibly other NER factors to the damage sites. This leads to more efficient lesion removal. Without Htz1 in the nucleosomes the higher acetylation levels on histone H3 cannot be achieved due to a less binding of Gcn5 to these nucleosomes, which results in reduced binding of Rad14 and, perhaps, other repair proteins to damaged DNA. Thereby, the lesion removal is compromised. This function of Htz1 cannot be replaced by canonical histone H2A because NER is compromised in both the *htz1Δ* strain and in the *swr1Δ* strain, which cannot deposit Htz1 to replace H2A/H2B in these nucleosomes.

NER is an essential DNA repair process that is required for genome stability in many organisms and that its absence in humans is linked to the cancer-prone condition xeroderma pigmentosum. Despite knowing the details of how NER operates on naked DNA *in vitro* (34) we are still unravelling the nuances of how it operates in the context of chromatin. Here we have demonstrated that its functionality within the yeast genome can be enhanced by the presence of the histone variant Htz1. This is the first evidence that a histone variant impinges on NER and links this histone variant to another important epigenetic event that governs NER efficiency, namely histone H3 acetylation.

## SUPPLEMENTARY DATA

Supplementary Data are available at NAR Online.

## FUNDING

UK Medical Research Council Programme Award (to R.W.); Career Development Fellowship from the Wellcome Trust (to C.B.M.). Funding for open access charge: UK Medical Research Council Programme Award.

*Conflict of interest statement*. None declared.

## Supplementary Material

Supplementary Data
