# Supplementary material for: Histone variant Htz1 promotes histone H3 acetylation to enhance nucleotide excision repair in Htz1 nucleosomes
Source: Nucleic Acids Res. 2013 Aug 7;41(19):9006–19. doi: 10.1093/nar/gkt688 (PMC3799447; doi:10.1093/nar/gkt688)
Supplement: Supplementary Data [file supp_41_19_9006__index.html]

Histone variant Htz1 promotes histone H3 acetylation to enhance nucleotide excision repair in Htz1 nucleosomes — Histone variant Htz1 promotes histone H3 acetylation to enhance nucleotide excision repair in Htz1 nucleosomes — Supplementary Data 

# Histone variant Htz1 promotes histone H3 acetylation to enhance nucleotide excision repair in Htz1 nucleosomes

## Supplementary Data

files

**Files in this Data Supplement:**

- Supplementary Data - pdf file
