# Supplementary material for: Histone variant Htz1 promotes histone H3 acetylation to enhance nucleotide excision repair in Htz1 nucleosomes
Source: Nucleic Acids Res. 2013 Aug 7;41(19):9006–19. doi: 10.1093/nar/gkt688 (PMC3799447; doi:10.1093/nar/gkt688)
Supplement: Supplementary Data [file supp_gkt688_nar-01201-d-2013-File010.pdf]

Supplementary Figure S1

A.

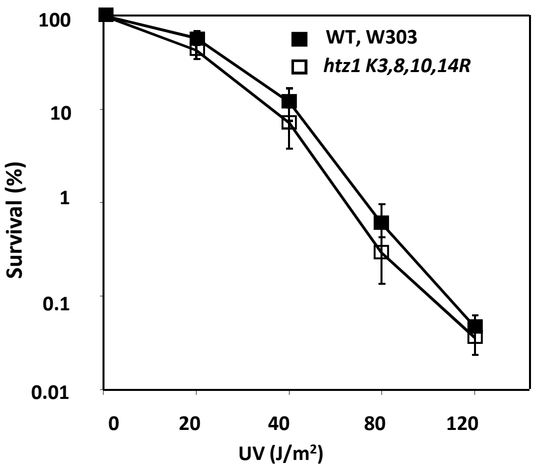

B.

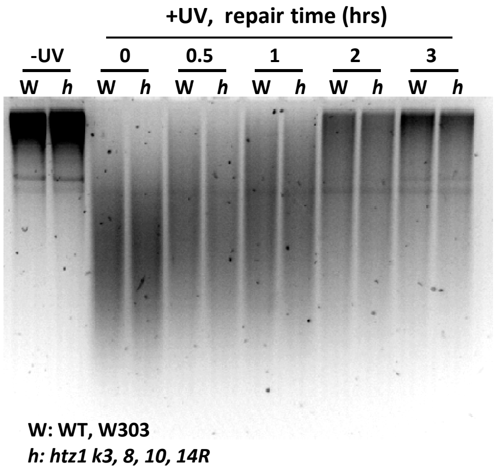

Figure S1. Htz1 acetylation at K3, 8, 10, 14 does not contribute to CPD repair and cell survival following UV irradiation. A The sensitivity to UV irradiation. In *htz1 K3,8,10,14R*, the four lysines at K3, 8, 10, and 14 of endogenous Htz1 were mutated to arginines (R). Data are the average of at least three independent experiments ± SD. **B** Repair of CPDs from the overall genome. DNA from samples taken before and after UV (100J/m^2) was treated with a CPD specific endonuclease. The digestion products were then separated by gel electrophoresis under denaturing conditions. The gel was stained with EtBr.

Supplementary Figure S2

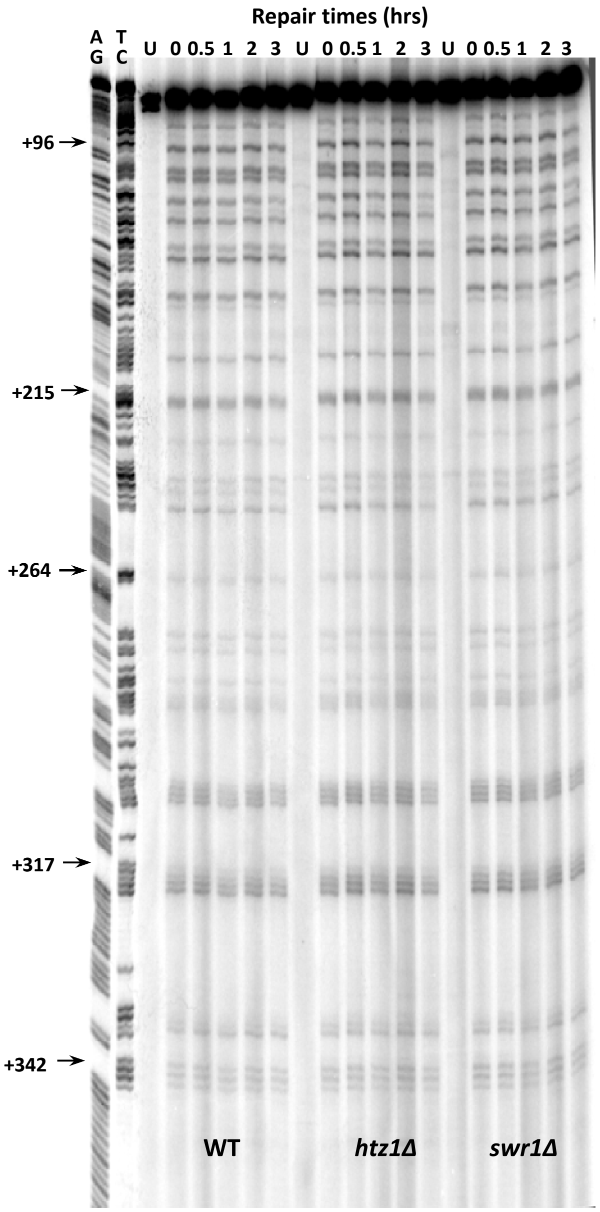

Figure S2. Repair of CPDs in the top strand of *HMRa1* coding region. Gel depicting CPDs in the top strand of the *Rsa*I-*Bgl*II fragment (+61 to +476) in the *HMRa1* sequence following a UV dose of 100 J/m^2. in the left of gel are the sequences of the strand of interest, shown in combinations of A, G and C, T, respectively. Lane U is DNA from mock irradiated cells while 0, 0.5, 1, 2 and 3 are DNA from irradiated cells following 0, 0.5, 1, 2 and 3 hour repair, respectively.

Supplementary Figure S3

A.

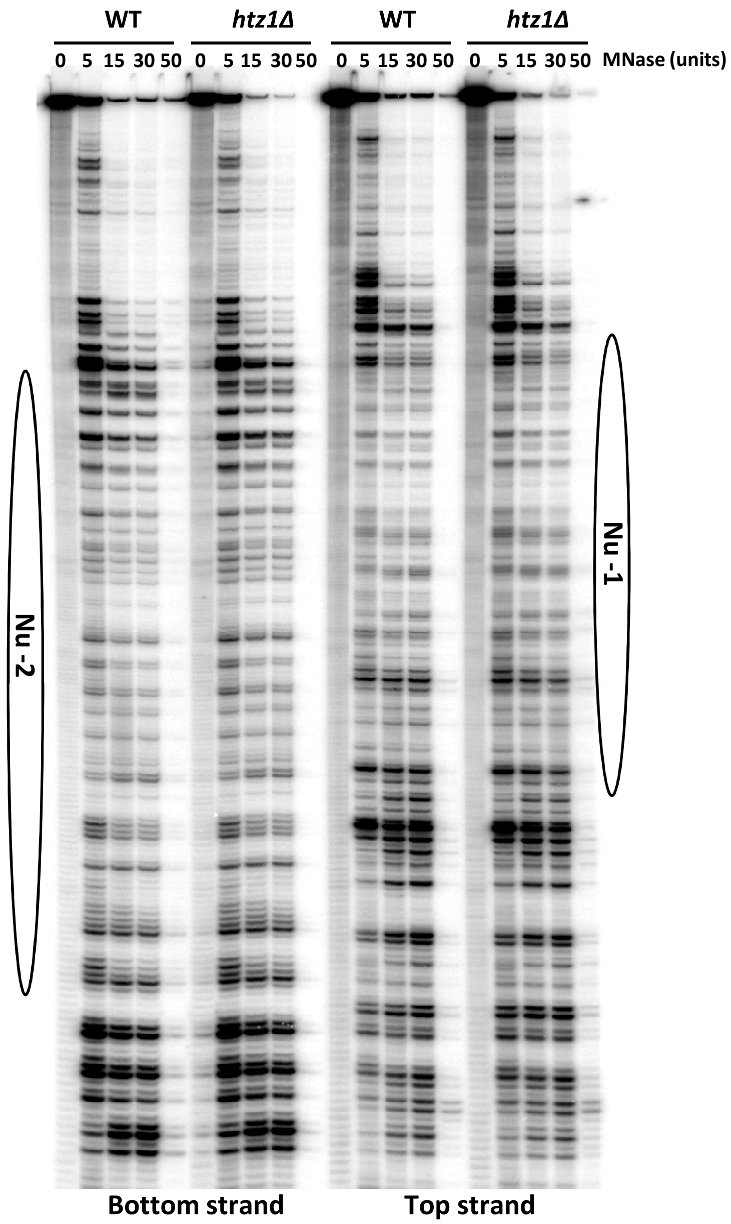

Supplementary Figure S3

B.

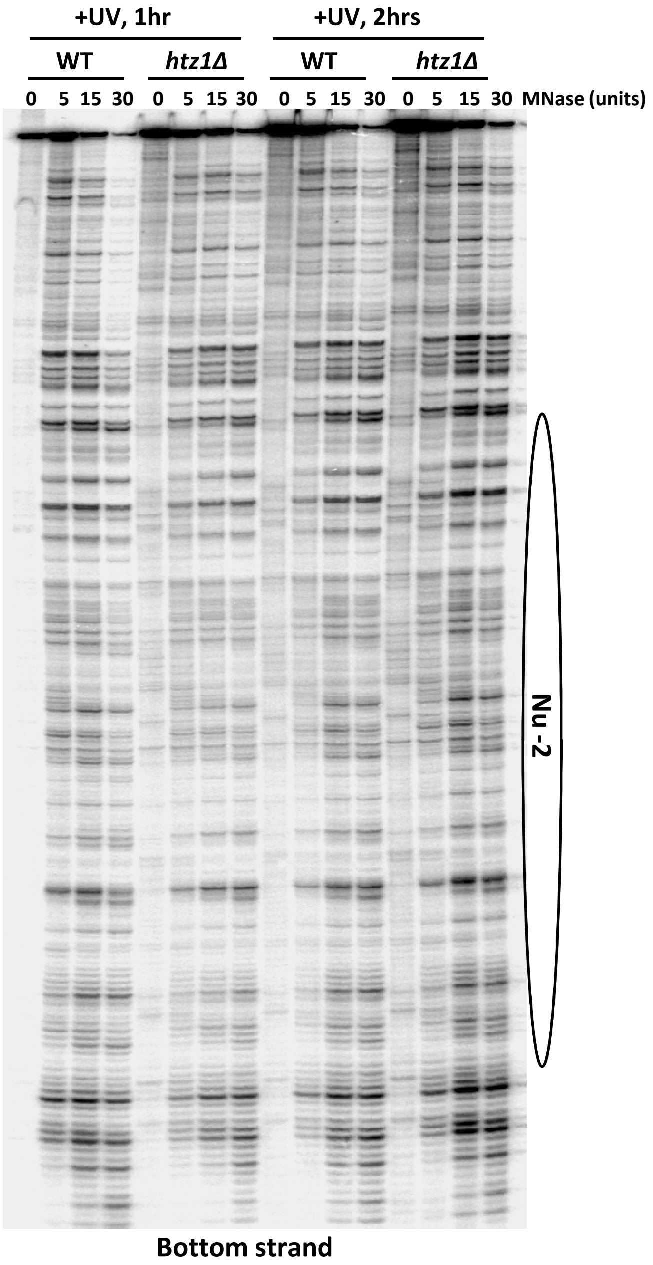

Supplementary Figure S3

C.

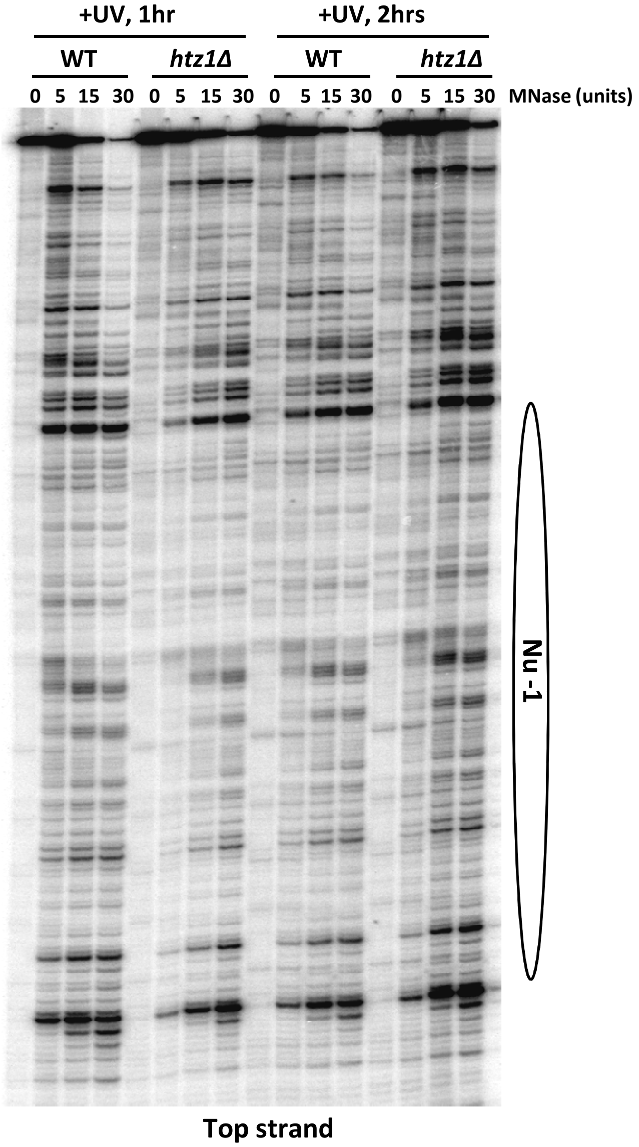

Figure S3. MNase sensitivity of the nucleosomal DNA in Htz1 nucleosomes in the *MFA2* promoter. Chromatin was extracted and treated with MNase as described in Materials and Methods. The region being analysed is the *HaeIII* fragment (-516 to +83) in the *MFA2* promoter. **A.** MNase sensitivity of the nucleosomal DNA in the *MFA2* promoter in wild type and the *htz1*Δ mutant without UV treatment. **B** and **C.** MNase sensitivity of the nucleosomal DNA in the *MFA2* promoter in wild type and the *htz1*Δ mutant after UV (100 J/m^2) and allowed repair times as indicated. The amount of MNase applied is indicated in units at the top of the gels.
